# Disentangling water sources in a gypsum plant community. Gypsum crystallization water is a key source of water for shallow-rooted plants

**DOI:** 10.1093/aob/mcab107

**Published:** 2021-08-18

**Authors:** Laura de la Puente, Juan Pedro Ferrio, Sara Palacio

**Affiliations:** 1 Departamento Biodiversidad y Restauración, Instituto Pirenaico de Ecología, Consejo Superior de Investigaciones Científicas, Avenida Nuestra Señora de la Victoria, 16, Jaca, ES-22700, Spain; 2 Unidad de Recursos Forestales, Centro de Investigación y Tecnología Agroalimentaria de Aragón (CITA), Av. Montañana 930, Zaragoza, ES-50059, Spain

**Keywords:** Water sources, hydrological niche, drought, gypsum crystallization water, plant community, root depth, gypsum affinity, water stable isotopes

## Abstract

**Background and Aims:**

Gypsum drylands are widespread worldwide. In these arid ecosystems, the ability of different species to access different water sources during drought is a key determining factor of the composition of plant communities. Gypsum crystallization water could be a relevant source of water for shallow-rooted plants, but the segregation in the use of this source of water among plants remains unexplored. We analysed the principal water sources used by 20 species living in a gypsum hilltop, the effect of rooting depth and gypsum affinity, and the interaction of the plants with the soil beneath them.

**Methods:**

We characterized the water stable isotope composition, δ ^2^H and δ ^18^O, of plant xylem water and related it to the free and gypsum crystallization water extracted from different depths throughout the soil profile and the groundwater, in both spring and summer. Bayesian isotope mixing models were used to estimate the contribution of water sources to plant xylem sap.

**Key Results:**

In spring, all species used free water from the top soil as the main source. In summer, there was segregation in water sources used by different species depending on their rooting depth, but not on their gypsum affinity. Gypsum crystallization water was the main source for most shallow-rooted species, whereas free water from 50 to 100 cm depth was the main source for deep-rooted species. We detected plant–soil interactions in spring, and indirect evidence of possible hydraulic lift by deep-rooted species in summer.

**Conclusions:**

Plants coexisting in gypsum communities segregate their hydrological niches according to their rooting depth. Crystallization water of gypsum represents an unaccounted for, vital source for most of the shallow-rooted species growing on gypsum drylands. Thus, crystallization water helps shallow-rooted species to endure arid conditions, which eventually accounts for the maintenance of high biodiversity in these specialized ecosystems.

## INTRODUCTION

Plant species from arid and semi-arid ecosystems have adapted to water scarcity by different mechanisms of water uptake and use. An important strategy is the segregation in hydrological niches, which makes possible the coexistence of different plant species in stable and diverse communities (Ehleringer *et al.*, 1991; Filella and Peñuelas, 2003; Araya *et al.*, 2011; Silvertown *et al.*, 2015, Palacio *et al.*, 2017). Hydrological niche segregation has often been found in several ecosystems affected by drought, such as Mediterranean shrublands and forests (Filella and Peñuelas, 2003; Moreno-Gutierrez *et al*., 2012; del Castillo *et al.*, 2016), deserts (Ehleringer *et al.*, 1991; Schachtschneider and February, 2010) or seasonal tropical forests (Liu *et al.*, 2010; Brum *et al.*, 2019; Ding *et al.*, 2021). Different traits related to changes in root architecture and rooting depth allow divergent water use strategies and the partition of this scant resource among coexisting plants (Donovan and Ehleringer, 1994; Moreno-Gutierrez *et al*., 2012). Water from precipitation present in the topsoil favours nutrient availability and microbial processes, using this pool preferentially during the growth period (Caldwell *et al.*, 1998; Querejeta *et al.*, 2021). However, during drought, roots should access deeper soil layers, sometimes even reaching the water table, where water availability is more stable (Ehleringer *et al.*, 1991; Ryel *et al*., 2008, 2010; Rempe and Dietrich, 2018). These deeper water pools are normally used to maintain transpiration during periods of limited growth (Voltas *et al.*, 2015). Many plants have developed dimorphic root systems with both superficial and deep roots, and the different water potential between dry shallow layers and wet deep layers can lead to hydraulic lift (Bauerle *et al.*, 2008; Prieto *et al*., 2012). This is a widespread process in semi-arid environments consisting of the passive movement of water from deeper layers to upper layers by roots (Prieto *et al.*, 2010, 2012). Through hydraulic lift, plants can act as ‘bioirrigators’ to neighbouring plants, hence increasing their chances of survival and, ultimately, the coexistence of diverse communities (Jackson *et al.*, 2000; Bayala and Prieto, 2020). Assessing the functional importance of contrasting soil water pools and their spatial and temporal variation is necessary to evaluate how climate change and land use may affect the ecohydrology of vegetation and the dynamics of plant communities (Ehleringer *et al.*, 1991; Oerter and Bowen, 2019; Dwivedi *et al.*, 2020). Understanding the mechanisms by which different plant species take up and partition water resources in arid and semi-arid conditions is crucial to unravel the processes supporting plant coexistence in dryland communites (Dodd *et al.*, 1998; Peñuelas *et al.*, 1999).

Gypsiferous soils, i.e. soils with high (>40 %) gypsum (Ca_2_SO_4_·2H_2_O) content (Casby-Horton *et al.*, 2015), are frequently present in drylands, being widespread around the world (FAO, 1990; Verheye and Boyadgiev, 1997). Together with the arid conditions, these soils have low water retention (Herrero and Porta, 2000) and, consequently, water is a major limiting factor for plants growing on gypsum soils. Some studies, however, found better water availability in summer in gypsum soils than in surrounding non-gypsum soils (Meyer and García-Moya, 1989; Escudero *et al.*, 2015). This observation is further supported by the discovery of crystalline gypsum water as a source for plants and other organisms during the dry period (Palacio *et al*., 2014b, 2017; Huang *et al.*, 2020). Gypsum contains water in its crystalline structure, which represents up to 20.8 % of its weight. Under certain conditions of vapour pressure and temperature (from 42 °C in pure gypsum, Marshall *et al.*, 1964), gypsum could dehydrate, changing into bassanite (the hemihydrate: CaSO_4_·0.5H_2_O) or into anhydrite (CaSO_4_) (Van’t Hoff *et al.*, 1903; Freyer and Voigt, 2003; Ossorio *et al.*, 2014). In addition, it has been demonstrated how this phase transformation can be induced by some micro-organisms, leading to anhydrite re-precipitation (Huang *et al.*, 2020). There is evidence of a large use of crystallization water by the gypsum endemic plant *H. squamatum*, and it has been suggested that its use could be extended to other shallow-rooted species living in gypsum plant communities (Palacio *et al*., 2014b, 2017; Henschel *et al.*, 2019). However, it is still unknown up to what point plants coexisting in the same plant community show different abilities to retrieve crystallization water, and thus whether the use of this water pool is a relevant factor defining hydrological niches in gypsum plant communities.

Gypsiferous soils also show particular chemical and physical properties, which could constrain the development of plant life (Escudero *et al.*, 2015). Plant roots have to cope with a high penetration resistance (Poch and Verplancke, 1997; Moore *et al.*, 2014, Sánchez-Martín *et al.*, 2021) and morphological transitions of the soil due to dissolution–precipitation sequences of gypsum (Casby-Horton *et al.*, 2015). In addition, most of these soils have a low nutrient supply caused by their low organic matter content and cation exchange capacity, and their saturation in Ca and S (Moore *et al.*, 2014; Casby-Horton *et al.*, 2015). Despite these limitations, gypsum soils host highly diversified floras, rich in endemic and highly specialized species (Moore *et al.*, 2014) which have been the subject of a more in-depth study from only a few years ago (Escudero *et al.*, 2015).

Plant species growing on gypsiferous soils can be classified into two groups depending on their affinity for gypsum: gypsophiles, which only grow on gypsiferous soils and often have substrate-specific physiological strategies (Palacio *et al.*, 2007; Escudero *et al.*, 2015; Cera *et al.*, 2021); and gypsovags, which are non-exclusive to gypsum soils (i.e. also grow off gypsum) and frequently display stress-tolerant strategies (Palacio *et al.*, 2007; Bolukbasi *et al.*, 2016). Gypsophiles have shown a range of mechanisms to detoxify the excess of Ca and SO_4_ considering their leaf elemental composition, whereas gypsovags would follow an avoidance strategy, reducing the absorption of these compounds (Palacio *et al.*, 2007, 2014a ; Merlo *et al.*, 2019; Cera *et al.*, 2021). Thus, if obtaining the crystallization water from gypsum is related to its dissolution (Huang *et al.*, 2020) and, consequently, the release of Ca and sulfate ions, gypsophiles could be more prone to using this water than gypsovags.

Tracing water movement in the soil and plants is possible using the natural variations of stable isotopes of hydrogen (^2^H) and oxygen (^18^O) in water molecules. This widely used method, extensively applied in hydrology and ecophysiology, allows evaluation of the result of several processes without disrupting the natural behaviour of the elements in the system (Meisner *et al.*, 2014; Penna *et al.*, 2018). Water phase changes (vapour–liquid–solid) explain most of the isotopic variability, as the heavier isotopes have a lower mobility (Dawson *et al.*, 2002). The water sources acquired by plants can be determined with the following premises (1) alternative water pools must be isotopically distinct and (2) there is no isotopic fractionation during water uptake. In dry environments, the first assumption is generally fulfilled: due to evaporative fractionation, upper soil layers often become enriched in the heavy isotopes ^2^H and^18^O, thus being distinguishable from deeper soil layers or groundwater (Barnes and Allison, 1988; Dawson and Ehleringer, 1998). With regard to the second assumption, fractionation during water uptake is considered negligible in most plants (Dawson *et al.*, 2002, and references cited therein), with the exception of some coastal wetland species (Lin *et al.*, 1993) and certain woody xerophytes (Ellsworth and Williams, 2007). Nevertheless, different authors have reported discrepancies between source and stem water, attributed to different causes, e.g. heterogeneity in the soil (Barbeta *et al.*, 2020), stem evaporation during periods of limited water flow (Dawson and Ehleringer 1993;Martín-Gómez *et al.*, 2015) or sampling artefacts (Marshall *et al.*, 2020)

The purpose of this study was to analyse the distribution of water sources among the 20 main dominant plant species in a hill top gypsum community. We characterized the variation in the isotopic composition of water along the soil profile and evaluated the effect of species rooting depth and affinity for gypsum soils on their water use in both spring and summer. We also analysed how plants interacted with the soil beneath them. Considering plant water uptake patterns, we hypothesized that: (1) shallow-rooted, gypsum-exclusive species will preferentially use crystallization water from gypsum in summer, whereas shallow-rooted, non-exclusive species will be restricted to the (scarce) free water available in the topsoil. Conversely, deep-rooted species, regardless of gypsum affinity, will rely mainly on the use of deep soil water and/or groundwater during summer drought. Considering plant–soil interactions, we also hypothesized that (2) deep-rooted species will interact with the shallow soil, lifting water up from deeper soil layers (hence performing hydraulic lift).

## MATERIALS AND METHODS

### Study area and species

We conducted field sampling on a gypsum hill in the Middle Ebro Depression, Zaragoza province, North-East Spain (1°37′52.5′′N, 0°41′23.7′′W, 287 m a.s.l) The main component of the soil in this region is gypsum (63.4 %), with thin outcrops of marls and clays (Quirantes, 1978). The climate is semi-arid and highly seasonal (Palacio *et al.*, 2007). Mean annual temperature is 14.9 °C, average annual rainfall is 331.5 mm, which falls mainly during spring and autumn, and evapotranspiration is around 1200 mm, so plants experience intense drought during summer months. An important proportion of the soil surface in the upper part of the gypsum hill is bare or coated with a biological crust dominated by cyanobacteria, lichens and mosses (Concostrina-Zubiri *et al.*, 2014). The plant community is dominated by sub-shrubs such as *Helianthemum squamatum*, with some taller shrubs, such as *Gypsophila struthium* subsp*. hispanica* or *Ononis tridentata* (Braun-Blanquet and Bolos, 1987).

We selected 20 dominant perennial plant species to represent the community living at the top of the hill, where stress conditions are most severe (Hodgson *et al.*, 1994; Guerrero Campo *et al.*, 1999; Casby-Horton *et al.*, 2015). These representative species included different life forms (woody vs. herbaceous), root depths, affinity for gypsum soils and taxonomic families. We considered species with roots >1 m deep to be deep-rooted species, and the rest were considered to be shallow-rooted (Guerrero-Campo, 1998; Table 1).

**Table 1.** Main characteristics of the study species.

Id	Species	Root depth	Maximum root depth	Gypsum affinity	Stem	Family
Fu.er	*Fumana ericifolia* Wallr.	Shallow	1–2	Gypsovag	Woody	Cistaceae
Ge.sc	*Genista scorpius* L.DC	Deep	3	Gypsovag	Woody	Fabaceae
Gy.hi	*Gypsophila struthium* L. subsp. *Hispanic*a (Willk.) G. López	Deep	3	Gypsophile	Woody	Caryophyllaceae
He.hi	*Helianthemum hirtum* (L.) Mill	Shallow	1–2	Gypsovag	Woody	Cistaceae
He.ma	*Helianthemum marifolium* (L.) Mill.	Shallow	1	Gypsovag	Woody	Cistaceae
He.sq	*Helianthemum squamatum* (L.) Pers.	Shallow	2	Gypsophile	Woody	Cistaceae
He.sy	*Helianthemum syriacum* (Jacq.) Dum.Cours.	Shallow	2	Gypsovag	Woody	Cistaceae
He.st	*Helichrysum stoechas* (L.) Moench subsp. *stoechas*	Shallow	2	Gypsovag	Woody	Asteraceae
He.fr	*Herniaria fruticosa* L.	Shallow	2	Gypsophile	Woody	Caryophyllaceae
Ko.va	*Koeleria vallesiana* (Honckeny) Gaudin subsp. *vallesiana*	Shallow	1–2	Gypsovag	Herbaceous	Poaceae
Le.su	*Lepidium subulatum.* L	Shallow	2	Gypsophile	Woody	Brassicaceae
Li.sf	*Linum suffruticosum* L.	Shallow	2–3	Gypsovag	Woody	Linaceae
Li.fr	*Lithodora fruticose* (L.) Griseb.	Shallow	2–3	Gypsovag	Woody	Boraginaceae
Ma.fr	*Matthiola fruticulosa* (Loefl. ex L.) Maire subsp. *fruticulosa*	Shallow	1–2	Gypsovag	Woody	Brassicaceae
On.tr	*Ononis tridentata* L.	Deep	3	Gypsophile	Woody	Fabaceae
Ro.of	*Rosmarinus officinalis* L.	Deep	3	Gypsovag	Woody	Lamiaceae
St.of	*Stipa offneri* Breistr.	Deep	3	Gypsovag	Herbaceous	Poaceae
Te.ca	*Teucrium capitatum* L. subsp*. capitatum*	Shallow	1–2	Gypsovag	Woody	Lamiaceae
Th.ti	*Thymelaea tinctoria* (Pourr.) Endl. subsp. *tinctoria*	Deep	3	Gypsovag	Woody	Thymelaeaceae
Th.vu	*Thymus vulgaris* L.	Shallow	2	Gypsovag	Woody	Lamiaceae

Maximum root depth: 1, 25–50 cm; 2, 50–100 cm; 3, > 100 cm (Guerrero-Campo, 1998).

### Plant and soil sampling

**Fig. 1.**
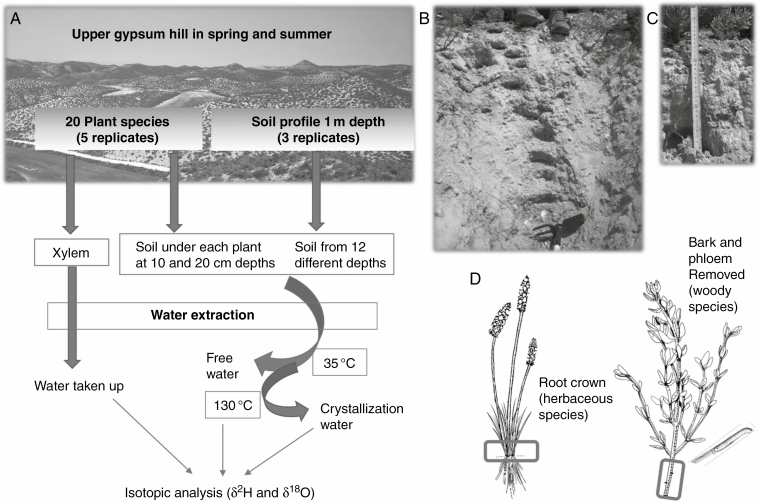
Overview of the sampling design. (A) Diagram showing the set-up for sample collection including replicate numbers and the subsequent extraction of water at different temperatures to obtain water samples for isotopic analysis. (B) Picture of one of the 1 m deep soil profiles. (C) Picture of soil collected underneath individual plants. (D) Description of plant sections used for xylem sampling, both in woody (removing the bark and phloem with a knife) and in herbaceous species (cutting and selecting the root crown).

Field sampling for isotope analyses was performed in the rainy spring (24–25 April, 2018) and in the dry summer (7–8 August, 2018), after a long rainless period. On each sampling date, we harvested the main stems (including the root crown) of five individuals of each species. We selected vigorous, medium-sized individuals located at least 5 m away from each other. To minimize the risk of stem water evaporation and to maximize the representativeness of xylem water as an indicator of the main water sources used by plants, we harvested between 06.30 and 10.00 h (solar time). In this time frame, we expect maximum transpiration rates and low evaporative demand to prevent stem dehydration (Grammatikopoulos *et al*., 1995; Martín-Gomez *et al*., 2017). In herbaceous species, the root collar was used as a proxy for non-enriched source water (Barnard *et al.*, 2006). In woody species, the bark and phloem were removed with a knife to avoid contamination with phloem water and organic compounds present in living cells and/or the bark (Ehleringer and Dawson, 1992; see Fig. 1D). Two soil samples were collected underneath each plant at two different depths: 10 and 20 cm, (approx. ± 2 cm) avoiding the intrusion of roots in the samples (see Fig. 1C) In addition, to capture variation in soil water isotopic composition throughout the soil depth, three profiles 1 m deep were dug underneath the bare soil on each sampling date (see Fig. 1B). Soil samples were collected at 12 different depths (approx. ± 2 cm): 5, 10, 15, 20, 30, 40, 50, 60, 70, 80, 90 and 100 cm. In spring, we gathered two extra samples from a small temporal creek upwelling in the saline depression downhill, representative of the groundwater. At the time of sampling, the water formed a small temporal creek, easily distinguished from rain puddles. Immediately after harvest, water, stem and soil samples were placed in airtight sealed tubes (Duran GL18), immediately frozen with dry ice and kept frozen until distillation in the lab.

### Water extraction

Xylem and soil water were extracted by cryogenic vacuum distillation (Ehleringer and Dawson, 1992), adapted as described in Palacio *et al*. (2014b). Spring samples were extracted at the Laboratory of Silvicuture of the Universitat de Lleida (Lleida, Spain) and summer samples were extracted with the same procedure at the laboratory of the Instituto Pirenaico de Ecología (IPE-CSIC, Zaragoza, Spain). Sample tubes were placed in a heated silicone oil bath, and connected with Ultra-Torr unions (Swagelok Company, Solon, OH, USA) to a vacuum system (approx. 10^–2^ mbar) including U-shaped water traps in series that were cooled with liquid nitrogen. Eight lines were installed. After an extraction time of 90 min for plant and soil samples (West, 2006; Meisner, 2014), captured water was transferred into screw-capped 2 mL vials, and stored at 4 °C until isotope analysis. Xylem water was distilled at 130 °C, whereas gypsum soils were distilled in two steps: first at 35 °C, and then at 130 °C to separate free and crystallization water and ensure almost complete dehydration of gypsum (Freyer and Voigt, 2003; Palacio *et al*., 2014b). Between the first and second distillation, sample tubes were kept in a desiccator with silica gel to avoid any re-hydration with ambient moisture, which could contaminate the next extraction water. Distilled samples were completely dried in the oven for 24 h at 60 °C. The samples were weighed before and after each distillation and after oven-drying to measure water content and confirm complete distillation.

### Stable isotope analyses

Oxygen and hydrogen isotope composition (δ ^18^O and δ ^2^H) were determined by cavity ring-down spectroscopy (CRDS). For spring samples, the analyses were performed at the Serveis Científico-Tecnics (Universitat de Lleida), using a Picarro L2120-i with vaporizer A0211 (Picarro, Santa Clara, CA, USA). Summer samples were analysed at the scientific services of the Instituto Pirenaico de Ecología (CSIC), using a Picarro L2130-i with vaporizer A0211. The estimated precision was 0.10 % for δ ^18^O and 0.40 % for δ ^2^H. Deuterium excess was calculated according to Dansgaard (1964), as the divergence from the Global Meteoric Water Line: Dex = δ ^2^H – 8 × δ ^18^O. Where appropriate, we applied the post-processing correction to manage the organic contamination of the samples. After describing the magnitude of contamination with the software PostProcess ChemCorrect™ v1.2.0, the H_2_^18^O, HD^16^O and H_2_^16^O peaks, filtered by the spectral features of organic compounds, were converted to organic-corrected δ ^18^O and δ ^2^H by applying a formula using device-specific factory calibration values (see Martín-Gómez *et al.*, 2015 for details).

### Statistical analyses

**Fig. 2.**
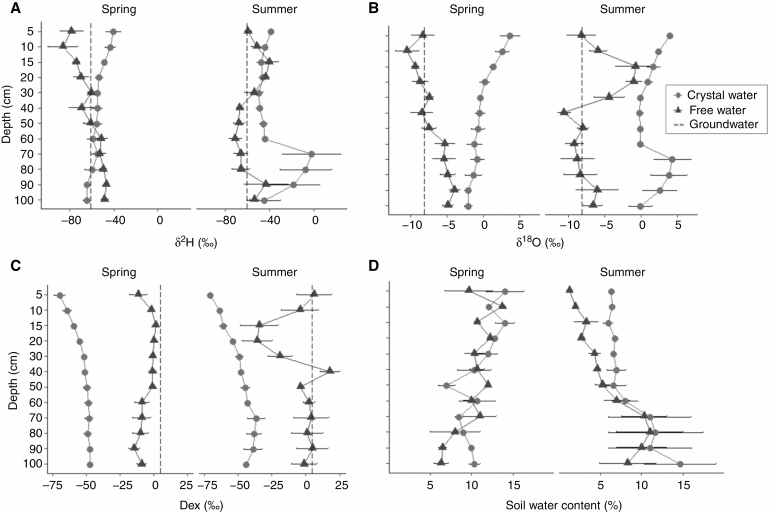
Changes in (A) mean δ ^2^H isotopic values, (B) mean δ ^18^O isotopic values, (C) mean water deuterium excess (Dex) values and (D) water content with depth along the soil profile in spring and summer. Black triangles indicate ‘free water’, extracted at 35 °C and grey circles indicate ‘crystallization water’ extracted at 130 °C. Values are means ± s.e. of the three bare soil profiles (*n* = 3). Dashed lines in (A–C) indicate groundwater isotopic values.

**Fig. 3.**
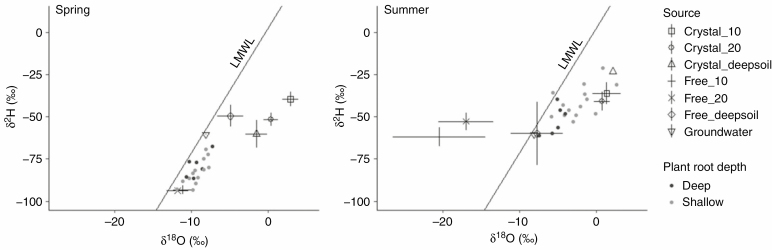
δ ^2^H and δ ^18^O composition of the xylem sap of the plant species and the seven different water sources used in Bayesian isotope mixing models. Water sources include: gypsum crystallization water extracted from the soil at 130 °C, free water extracted from the soil at 30 °C and groundwater. Soil from 10 and 20 cm deep was sampled underneath each plant, deep soil was sampled in the profiles and groundwater was upwelling in saline depressions in spring. Grey points indicate shallow-rooted plants and black points indicate deep-rooted plants. LMWL: local meteoric water line.

Changes in soil water content and in the isotopic composition of water along soil profiles, as well as δ ^2^H–δ ^18^O bi-plots with soil water and xylem sap isotopic compositions were visualized using ggplot2 in R (Wickham, 2016). Soil water content was calculated from sample weights before and after water extractions. Variation in the isotopic composition along the soil profiles was analysed to characterize potential deep water sources for plants and locate the evaporation front in both seasons. To identify the possible sources of deep soil water for plants, we defined soil depths >20 cm with homogeneous isotopic composition of free soil water that markedly differed from other depths in the soil (Fig. 2). Transition depths with intermediate and highly variable soil water isotopic composition were not included in the model, so that alternative sources could be clearly differentiated. For this reason, water isotopic values at 30 and 40 cm depth were removed from the set of sources (see Fig. 2 and Supplementary data Fig. S1). Considering the results for the characterization of soil water along the soil profile (see Figs 2 and 3), we could differentiate four potential water sources for plants (see below). This characterization of sources was the simplification of a preliminary, seven-source model (see below).

Differences among study species and sampling dates in xylem water isotopic composition (δ ^18^O and δ ^2^H) and deuterium excess were evaluated using residual maximum likelihood (REML) analysis with the lmer function from the lme4 package in R (Bates *et al.*, 2015). Models were run separately for each water isotope, ^2^H, ^18^O and deuterium excess. General models included species nested within family as a random factor to account for potential phylogenetic bias, and gypsum affinity (two levels: gypsophile and gypsovag), water pool (two levels: free and crystallization) and root depth (two levels: shallow and deep) as fixed factors. Separate models were run for each season to explore differences between species with different gypsum affinity and root depth in each season for each isotope. Shapiro–Wilk normality test (Royston, 1995) and Levene test for homogeneity of variances (Noguchi and Gel, 2010; Gastwirth *et al.*, 2009) were used to check the normal distribution and homoscedasticity of residuals. Residuals were visually checked using the DHARMa package (Hartig, 2021). When interactions were significant, groups were analysed with post-hoc Tukey HSD tests using the lsmeans package (Russell, 2016).

The relative contribution of different water sources to xylem sap was estimated using Bayesian mixing models for stable isotopic data with the package MixSIAR (Stock *et al.*, 2018). This procedure estimates the proportion of source contributions to a mixture. The model used as ‘consumers’ the isotope values of xylem water in each individual (δ ^2^H and δ ^18^O). For ‘sources’, alternative models were run with different grouping of sources in order to select those that best described and simplified the potential water sources for plants. The Mix-SIAR model, that had better accuracy and so explained better the contribution of the sources to the xylem of plants, was run with seven different sources for each species: free soil and crystallization water from 10 and 20 cm, free and crystallization water from the ‘deep soil’ (50–100 cm combined) and groundwater. Values for 10 cm and 20 cm soil depth included one replicate per individual plant, whereas values from deeper soil were averaged across the three soil profiles. This model was simplified *a posteriori* by the addition of the contributions of each source into four simplified sources: (1) ‘crystal water’, i.e. gypsum crystallization water from the soil underneath the plants and deep soil, as they clearly departed from free water, and had a comparatively small variation along the soil profile. It was calculated by the addition of the contributions to the xylem of plants of all three crystallization water sources initially considered. (2) ‘Shallow free’, i.e. free water in the shallow soil (until approx. 20 cm depth), represented by free water extracted from soil collected underneath each plant owing to the better replication. It was calculated as the addition of the contribution to the xylem of the free water at 10 and 20 cm. (3) ‘Deep soil free’, i.e. free water in the deep soil (between 50 and 100 cm depth). (4) The water table (i.e ‘groundwater’), not modified from the output in the Bayesian model. The contribution of the water sources to the species separated by their root depth was calculated by the addition of the contributions of the different sources to the composition of the xylem water of the different species in each rooting depth group.

The effect of plant species on the isotopic composition of the soil beneath them was considered by assessing the significance of between- and within-group variations in the isotopic composition of the soil collected under each individual. Effects were analysed separately for each isotope (δ ^2^H, δ ^18^O and deuterium excess) and season. To account for interspecific differences in the isotopic composition of soil water, we ran lineal models using the lm function (Chambers, 1992). Specific models were run with REML using the lmer function (lme4 package) to assess differences for the fixed factors: ‘gypsum affinity’, ‘root depth’ and their interaction with the same random term structure as in xylem water comparisons. To assist in the interpretation of plant–soil interactions, e.g. to visually identify evidence of hydraulic lift, the isotopic compositions of the xylem water and the water extracted from the soil beneath the plants were visually compared using the ggplot2 package. All statistical analyses were run in R 4.0.0. (R Core Team, 2020).

## RESULTS

### Water source characterization along soil profiles

δ ^2^H and δ ^18^O composition of free soil water showed more homogeneous values in spring than in summer (Fig. 2A, B), mainly due to the spatial heterogeneity of soil water evaporative enrichment and the location of the evaporation front at slightly different positions among the three different soil profiles. In spring, water in shallow soil layers showed more negative values of both δ ^2^H and δ ^18^O than water in the deep soil (Fig. 2A, B), which corresponded to very negative values from a recent rain event in April 2018 (Supplementary data Table S1). No evaporation front was observed in spring, whereas, in summer, the evaporation front in the bare soil was located at approx. 15 cm depth, showing an abrupt change from isotopically depleted values of δ ^2^H and δ ^18^O at 5–10 cm, typical of water vapour, to highly enriched values at 15–20 cm (Fig. 2A, B). Below 20 cm, δ ^2^H and δ ^18^O became more negative with depth, until they stabilized from 40–50 cm to 80–90 cm depth, with a slight increase from 90 to 100 cm (Fig. 2A, B). In both seasons, the δ ^2^H and δ ^18^O of gypsum crystallization water showed a similar pattern with depth (between 5 and 60 cm). Values were more positive in the upper soil layers, presumably due to the re-crystallization of gypsum with more evaporated water. In spring, this progressive depletion with depth continued until 100 cm, whereas, in summer, a small increase in isotopic signatures was observed between 70 and 90 cm, together with larger variability among profiles.

In spring, deuterium excess of free water was rather homogeneous throughout the soil profile (Fig. 2C), becoming slightly negative in the top layer (5 cm) and in the deepest layers (60–100 cm). Conversely, deuterium excess of free water in summer showed large variations, following an opposite pattern to that in δ ^2^H and δ ^18^O that indicates strong evaporative enrichment of soil water in the upper soil layers (Fig. 2C). For crystallization water, deuterium excess in both seasons became less negative with depth, further indicating re-crystallization of gypsum with more evaporated water in the top soil layers.

We found much higher free water content in the shallow soil layers in spring than in summer (Fig. 2D). In spring, we observed relatively uniform free water content in the soil profile until 60–70 cm, where soil water content decreased in the vicinity of the underlying bedrock. In summer, we observed severe soil desiccation in shallow soil layers and higher water content with depth, until reaching layers next to the bedrock, where the soil water content decreased again. The content of crystallization water retrieved is related to the gypsum content in the soil which was homogeneous through most of the soil profile in summer. Nevertheless, we found more variability in the upper layers in spring (Fig. 2D).

Regarding the position of the water sources and the xylem of plants in the bi-plot showing δ ^2^H vs. δ ^18^O, we observed the segregation of crystallization and free water, and the clustering of the xylem sap of shallow-rooted plants with crystallization water during summer. This is compatible with an important use of this water source by these species during drought (see below). Free water from the first 20 cm in the soil (collected underneath the plants) showed values typical of water vapour (Fig. 3; Supplementary data Fig. S2). Contrastingly, free water collected underneath the bare soil, which retained more water, showed values of evaporated water (Fig. 2; see Supplementary data Fig. S2). These could be due to the biological and physical crust formed in the bare soil that decreases evaporation (Escudero *et al.*, 2015) and/or to the more intense exploitation of the scarce free water from the soil beneath them by plants. Further, many of the isotopic values of shallow-rooted plants with a high gypsum water contribution in their xylem sap cannot be solely explained by an eventual evaporation within the stem (see Supplementary data Fig. S3).

### Analysis of factors explaining differences among plants in their xylem isotopic composition

**Fig. 4.**
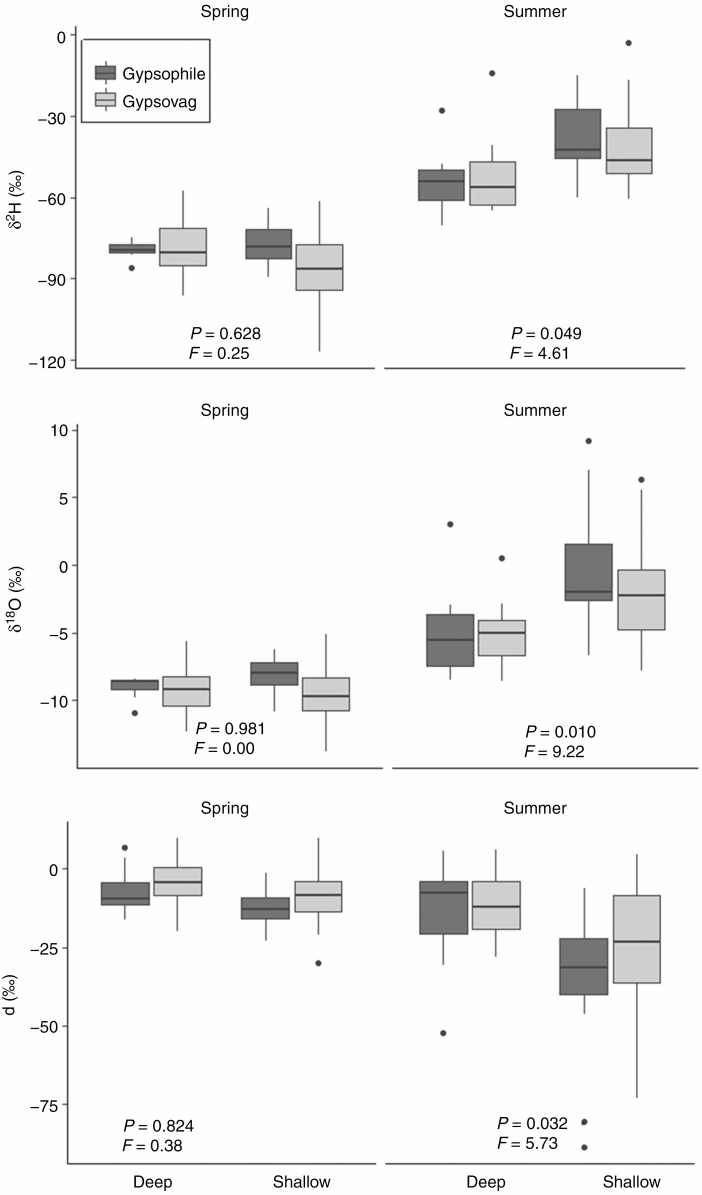
Seasonal variation in the isotopic composition of xylem water, according to root depth (spring, left panels; summer, right panels). δ ^2^H and δ ^18^O isotope composition and deuterium excess are shown. Different letters indicate significant differences after Tukey’s post-hoc analyses across root depth and season (*P* < 0.05). *F*-ratios and *P*-values show differences in the xylem sap between plants with distinct root depth, in models run separately for each season, Black boxes indicate gypsophiles and grey boxes gypsovags.

Both season and rooting depth had a significant effect on the isotopic composition of the xylem water of the target species. Conversely, the affinity for gypsum soils did not show a significant effect on xylem water composition, indicating that plants did not use different water sources according to this factor. Three main groups could be identified based on their xylem water composition: the first group included all species in spring, whereas the second and third groups included summer values for shallow-rooted and deep-rooted species, respectively (Fig. 4; Supplementary data Table S2). Differences in the isotopic composition of the xylem water of plants were highly significant between seasons, as well as for the interaction between season and root depth. In spring, δ ^2^H and δ ^18^O had more negative values than in summer, and more positive deuterium excess, but the xylem sap isotopic composition did not show significant differences due to species rooting depth. In summer, however, deep-rooted species had more negative values than shallow-rooted species (Fig. 4; Supplementary data Table S3). Overall, these results indicate that in spring all plants in the community used similar water pools, whereas in summer plants used different water sources, depending on their rooting depth, and irrespective of gypsum affinity.

### Contribution of different water sources to the xylem water of plants

**Fig. 5.**
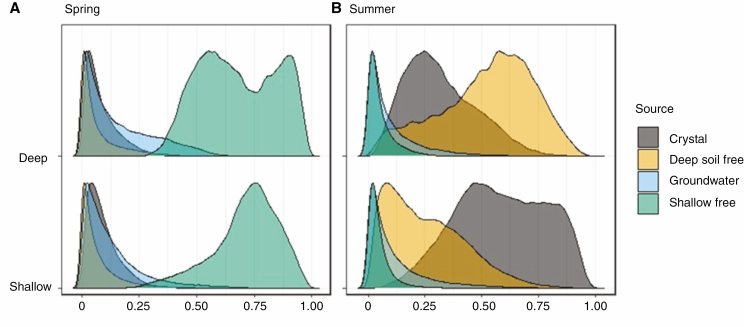
Results from Bayesian stable isotope mixing models showing the estimated contribution of different water sources, namely: shallow free water (10–20 cm), deep free water (50–100 cm), groundwater and gypsum crystallization water (all depths combined) to the xylem water of 20 dominant plants from a gypsum hill top community, grouped into deep-rooted and shallow-rooted species.

Estimation of the most likely sources of water used by plant species by Bayesian models revealed that, in spring, all plants used a large proportion of free water from the shallow soil (estimated using 10–20 cm underneath the plants). However, in summer, crystallization water from gypsum was the main source for shallow-rooted species, whereas deep soil water (50–100 cm) was the main source for deep-rooted species (Fig. 5; Supplementary data Fig. S4). In spring, we also detected a moderate contribution of groundwater (16 % for deep-rooted and 13 % for shallow-rooted), particularly in the deep-rooted *Ononis tridentata*, *Gypsophila hispanica* and *Genista scorpius*, and the shallow-rooted *Teucrium capitatum*, *Hernaria fruticosa* and *Fumana ericifolia* (Supplementary data Fig. S4). In summer, the main source of water for shallow-rooted plants was crystallization water (59 %), irrespective of species affinity for gypsum soils. In addition, 30 % of the water used by shallow-rooted plants was free soil water from deeper layers (50–100 cm; Fig. 5; Supplementary data Figs S4 and S5). Deep-rooted species in summer mainly used free water from the deeper soil layers (52 %), but crystallization water still accounted for 32 % of the water used by these plants (Fig. 5; Supplementary data Figs S4 and S5).

### Soil–plant interaction

In spring, soil underneath the plants (10–20 cm depth) showed significant species-specific variations in δ ^2^H and δ ^18^O for both free and crystallization water, and in deuterium excess for crystallization water (Table 2). We also found significant differences among species in summer, for free water δ ^18^O and deuterium excess (Table 2). In summer, we did not find significant effects of either rooting depth, gypsum affinity or their interaction on the isotope composition of soil free water collected beneath plants (Supplementary data Table S4). Free water isotopic composition of the shallow soil beneath some of the deep-rooted species (*G. scourpius*, *G. hispanica*, *Rosmarinus officinalis* and *Thymelaea. tinctoria*) during summer was similar to their xylem water isotopic composition for δ ^2^ H, and closer to that of deep soil layers than in other species, providing indirect evidence of hydraulic lift by these species. However, the δ ^18^ O composition of the soil was consistently more negative than the xylem isotopic composition of plants (Supplementary data Fig. S6).

**Table 2.** Results of linear models analysing the effects of species on the isotopic composition (δ ^18^O and δ ^2^H) of soil free water collected underneath the plants (10–20 cm depth).

Season	Isotope	F	P-value
Spring	δ ^2^H	3.80	**<0.001**
	δ ^18^O	3.54	**<0.001**
	D-ex	1.18	0.279
Summer	δ ^2^H	1.33	0.173
	δ ^18^O	3.05	**<0.001**
	D-ex	3.09	**<0.001**

*F*-values and *P-*values are shown. Bold type indicates significant effects at *P* < 0.05.

D-ex, deuterium excess.

## DISCUSSION

Our results agree with previous studies that demonstrate the role for summer drought as a structuring factor in plant communities growing on gypsum drylands (Palacio *et al.*, 2017). Hydrological niche segregation differentiates functional strategies between shallow-rooted species, dominant in these communities, and deep-rooted plants. This spatial segregation could have consequences on plant community assembly, promoting diverse plant communities whose variable response to soil moisture decrease enhances their stability under arid conditions (Peñuelas *et al.*, 2011; Silvertown *et al*., 1999, 2015).

We identified gypsum crystallization water as a crucial component of the water balance in gypsum drylands. Water held in the crystalline structure of gypsum was the most important water source for almost all shallow-rooted species and a highly relevant water source for deep-rooted species during summer drought. Our results demonstrate that gypsum crystallization water is widely used by plants, irrespective of their affinity for gypsum soils. Contrary to our predictions, both gypsum-endemic and non-endemic species (gypsophiles and gypsovags) with shallow roots used gypsum crystallization water as the preferential water source during summer. The uptake mechanisms that make such use possible remain undescribed. The similar isotopic composition of gypsum crystallization water in both seasons agrees with the notion that continuous processes of gypsum dissolution–precipitation take place during the year, involving both precipitation and more evaporated free soil water (Fig. 2; Van Driessche *et al.*, 2012). It is known that the temperature for pure gypsum dehydration can be decreased by some ionic solutions (Gázquez *et al.*, 2017). Recent findings indicate that some micro-organisms can dissolve gypsum rock by secreting organic acids, retrieving crystallization water under extreme xeric conditions (Huang *et al.*, 2020). We suggest that plant roots and their associated micro-organisms could similarly be altering gypsum to mine its crystalline water. This is supported by several previous studies providing evidence on the ability of plants and their associated micro-organisms to exude organic acids and other compounds that alter the substrate where they grow (Bashan *et al.*, 2002; Puente *et al.*, 2004; Chaparro *et al.*, 2013; Lebre *et al.*, 2017). However, detailed analyses on the specific compounds that plants could be secreting to the gypsum soil, and their potential effect on the thermodynamic equilibrium among gypsum phases, are lacking.

Other studies identified groundwater as the main water source enabling the maintenance of activity during drought for deep-rooted species (Salvucci and Entekhabi, 1995; Fan *et al.*, 2017; Koirala *et al.*, 2017; Palacio *et al.*, 2017). In contrast, our results indicated water from 50–100 cm depth (i.e ‘rock moisture’, Rempe and Dietrich, 2018) as the main water source in summer for deep-rooted species in the studied community. Although its dynamics and hydraulic properties have not yet been explored in detail (Dwivedi *et al.*, 2020), this crucial source of water probably came from precipitation that passed through unsaturated weathered bedrock until reaching the groundwater (Oshun *et al.*, 2016; Rempe and Dietrich, 2018). Despite the isotopic composition of groundwater and deep soil water being very similar in summer, for consistency between the spring and summer models, we kept the same water sources in the Bayesian models for both seasons. The model choice for the deep free water instead of groundwater could probably be due to its higher variability and higher probability area. Although we cannot untangle the use of these sources by plants during summer, groundwater did not outflow in the creek located under the study hill during summer (L. de la Puente, pers. obs.), being sited >10 m deep from the top of the hill. Consequently, considering the plants’ position at the top of a hill and their observed (relatively limited) root length, deep soil free water seems a more plausible source of water for these plants than groundwater. Plants may also show a preference for rock moisture over groundwater, as happens with large trees that take advantage of the oxygenated conditions of the weathered bedrock (Zwieniecki, and Newton, 1996; Graham *et al.*, 2010; Liu *et al.*, 2014; Hahm *et al.*, 2020). Interestingly, our results show that not only deep-rooted species, but also some relatively shallow-rooted species (*Teucrium capitatum*, *Linum suffruticosum* and *Lithodora fruticosa*), were mainly using free water from the deeper soil during summer (Supplementary data Fig. S2). The maximum rooting depths of these species is between 50 and 100 cm depth (Guerrero-Campo, 1998), with actual rooting depth being sensitive to reach free water (Hodge, 2004; Fan *et al.*, 2017). Nevertheless, we cannot rule out the possibility that these plants could also be using free water from slightly shallower layers, i.e. 30–40 cm deep, which had an isotopic composition similar to that from 50–100 cm deep, but was not included in the Bayesian models due to its variability and slight similarity with water from 20 cm depth. In any case, the use of free water by these species could be favoured through the segregation of water sources between coexisting shallow-rooted species to mitigate competition. Further approaches comprising experimental manipulation of resources or models to determine the processes that stabilize community composition best would be required to ascertain these possibilities (Stoll and Weiner, 2000; Silvertown *et al.*, 2015).

Another explanation for the use of deep soil water from relatively shallow rooted plants might be the hydraulic lift by some deep-rooted species during summer. The species *Genista scorpius*, *Gypsophila struthium* subsp. *hispanica*, *Rosmarinus officinalis* and *Thymelaea tinctoria* showed similar δ ^2^H isotopic values between the shallow soil beneath them and their xylem composition (Supplementary data Fig. S4). This indicated water drawing up from the deeper soil, which could also be available to neighbouring shallow-rooted species. According to previous studies considering the composition of just one of the water stable isotopes (δ ^2^H or δ ^18^O) (Dawson, 1993;Ludwig, 2003; Durand *et al.*, 2007) to prove this phenomenon, we could have indirect evidence of hydraulic lift in the dry season in our system. Nevertheless, further investigations including information on the water used by shallow-rooted plants located close to deep-rooted species potentially drawing up water are required to prove the influence of hydraulic lift by deep-rooted plants on neighbouring shrubs (Filella and Peñuelas, 2003).

We observed a significant effect of plant species on the isotopic composition of the free water from the soil beneath them in spring, when plants were using water available in the shallowest soil layers (10–20 cm). This suggests that the microenvironment created under plants is species specific and is able to modify soil water conditions. In summer, we observed an effect of the species on the δ ^18^O isotopic composition and deuterium excess of free shallow water, but not for free water δ ^2^H. This could be due to a pore scale isotope heterogeneity in the water soil caused by water surface interaction effects (Penna *et al.*, 2018) or to the differences in the relative contribution of equilibrium and kinetic effects during evaporative enrichment for δ ^18^O and δ ^2^H, which cause different sensitivity to environmental variables (Craig and Gordon, 1965; Cappa *et al.*, 2003). Recent meta-analysis on the environmental drivers of leaf water isotopic composition revealed that δ ^2^H is more related to the isotopic composition of source water and atmospheric vapour, whereas δ ^18^O seems to be more responsive to relative humidity of the air (Cuntz and Cernusak, 2020). Extrapolating these processes to the soil, it is reasonable to expect more homogeneous δ ^2^H isotopic values in the soil during summer, whereas δ ^18^O isotopic values would be more variable owing to the different soil microenvironment during evaporative enrichment underneath each species.

### Conclusions

To conclude, our results prove that during drought there is a partitioning of water sources among coexisting species, which segregated the species’ hydrological niche by root depth, but not by gypsum affinity. In this plant community living on the top of a gypsum hill, crystallization water of gypsum represents a vital source for most of the shallow-rooted species during summer, and allows them to survive the arid conditions, forming diverse communities. Rock moisture arises as the main water source for deep-rooted species during drought. However, our results show that all species in the community are able to use crystalline gypsum water during the summer drought period, indicating a hidden water pool important for life in gypsum drylands. Hence, we strongly recommend that gypsum crystallization water is included as a potential source in water balance studies dealing with ecosystems developed on gypsum soils, which span >200 Mha in all continents (Eswaran and Gong, 1991).

## SUPPLEMENTARY DATA

Supplementary data are available online at https://academic.oup.com/aob and consist of the following. Table S1: precipitation isotopic values in Zaragoza. Table S2: results of GLMM analysing the effects of the root depth, affinity for gypsum soils, season and their interaction on the isotopic composition of the xylem water of plants. Table S3: results of GLMMs analysing the effects of root depth, affinity for gypsum soils and their interaction on the isotopic composition of the xylem water of plants in spring and summer. Table S4: results of GLMMs analysing the effects of root depth and gypsum affinity on the isotopic composition of the soil free water underneath plants. Figure S1: δ ^2^H and δ ^18^O biplot for xylem values and sources including free and crystallization water between 30 and 40 cm deep. Figure S2: summer water content in the first 10 and 20 cm of the bare soil and soil underneath the plants. Figure S3: isotopic composition of the soil up to 50 cm and of the shallow rooted plants in summer. Figure S4: contribution of the four main water sources to the xylem water of each species in both seasons. Figure S5: contributions of seven different water pools included in Bayesian stable isotope mixing models to the xylem of deep- and shallow-rooted species in both seasons. Figure S6: summer isotopic values of free water underneath plants and their xylem water.

mcab107_suppl_Supplementary_Materials

## Data Availability

The raw RNA-Seq data generated in this study are available from the corresponding author upon request.
