# Supplementary material for: Disentangling water sources in a gypsum plant community. Gypsum crystallization water is a key source of water for shallow-rooted plants
Source: Ann Bot. 2021 Aug 18;129(1):87–100. doi: 10.1093/aob/mcab107 (PMC8829898; doi:10.1093/aob/mcab107)
Supplement: mcab107_suppl_Supplementary_Materials [file mcab107_suppl_Supplementary_Materials.docx]

**
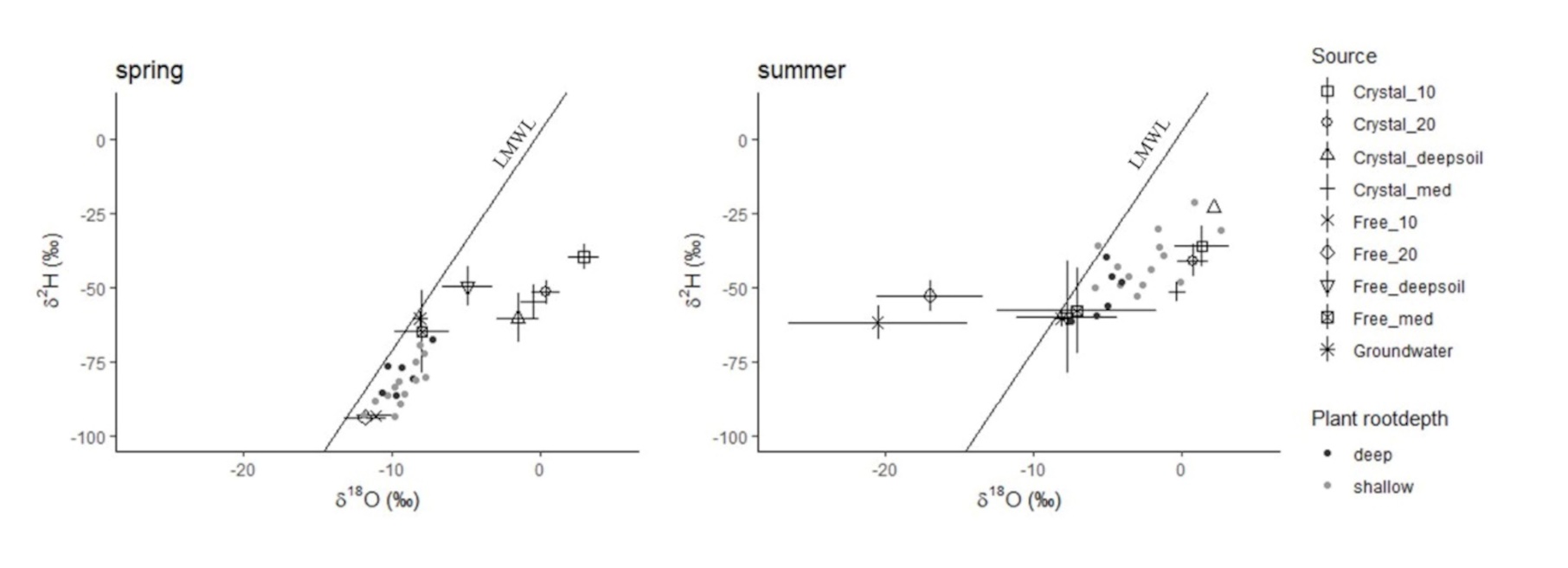
**SUPPLEMENTARY DATA

**Fig. S1.** δ^2^H and δ^18^O composition of the xylem sap of the plant species and eight different water sources. Water sources include gypsum crystallization water (“Crystal“) and free water (“Free“) in the soil at different depths. 10 and 20 cm deep soil was collected under the plants and deeper soil was collected from the profiles. “med” represents the mean composition of the water extracted from the soil at 30 and 40 cm deep, and “deepsoil” represents a mean composition of the soil from 50 to 100 cm deep. Grey points are for shallow rooted plants and black points for deep rooted plants. Groundwater is also represented. LMWL: local meteoric water line


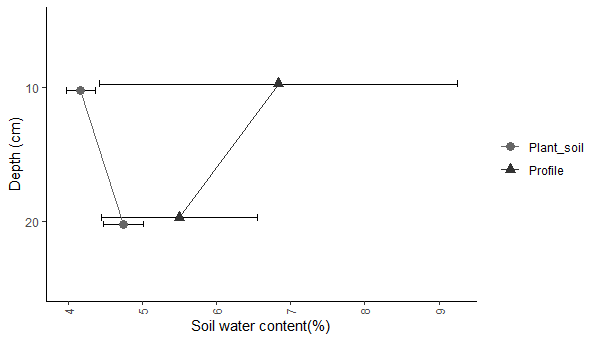


**Figure S2**. Summer water content in the first 10 and 20 cm of the soil. Grey points show values for the water underneath the plants and black triangles the values in the profiles (bare soil).


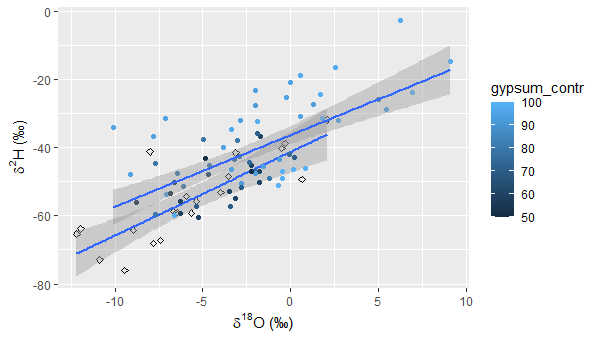


**Figure S3** Isotopic composition of the free water in the bare soil up to 50 cm deep, obtained from the soil profiles (empty diamonds) and isotopic composition of the xylem of shallow rooted plants in summer (filled circles). The colour scale of the points represents gypsum water contribution in the species xylem sap according to the MixSIAR model. Lighter blue for larger contribution and darker blue for smaller contribution.

**
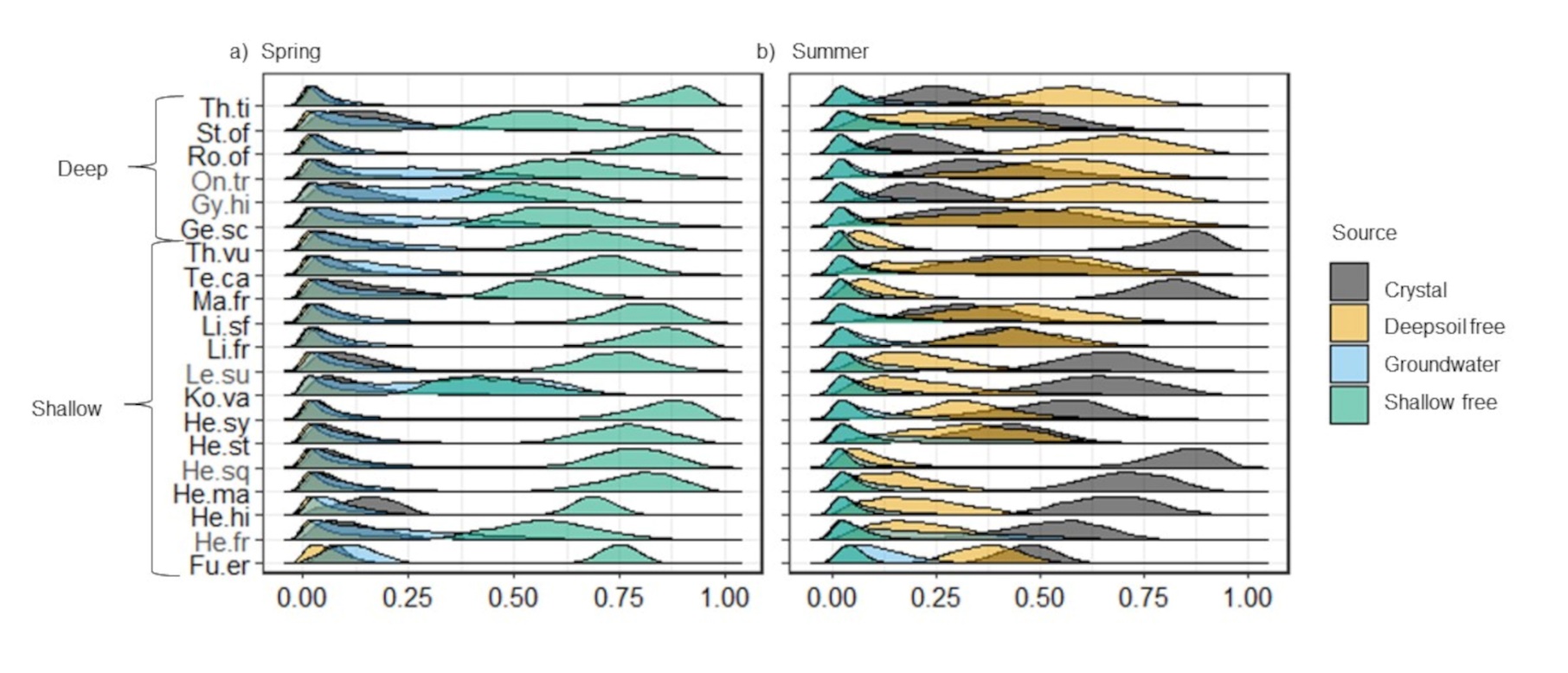
Figure S4.** Results from Bayesian stable isotope mixing models showing the estimated contribution of shallow free water (10 – 20 cm), deep free water (50 – 100 cm), groundwater and gypsum crystallization water (all depths combined) to the xylem water of 20 dominant species coexisting in a gypsum hill in NE Spain. The first 6 species were deep-rooted, and the following fourteen were shallow-rooted. Gypsovags are labelled in black, gypsophyles in grey.


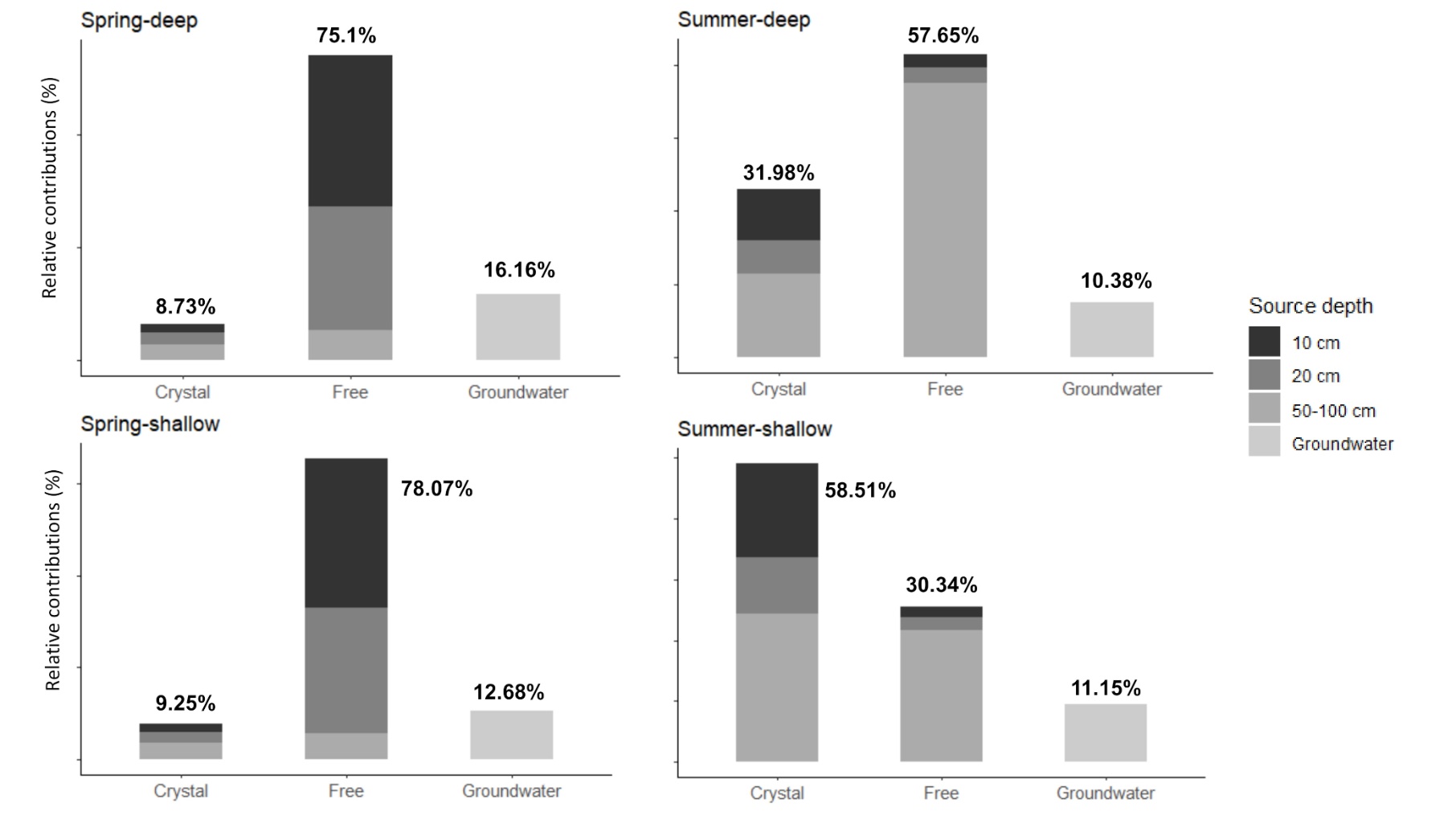
**Figure S5.** Results from Bayesian stable isotope mixing models showing the contribution of seven different water pools to the xylem water of plants, analysed separately for deep- and shallow-rooted species in each season. Percentages show the total contribution of gypsum crystallization water (crystal), free soil water and groundwater to the xylem of the shallow and deep rooted species, in spring and summer.


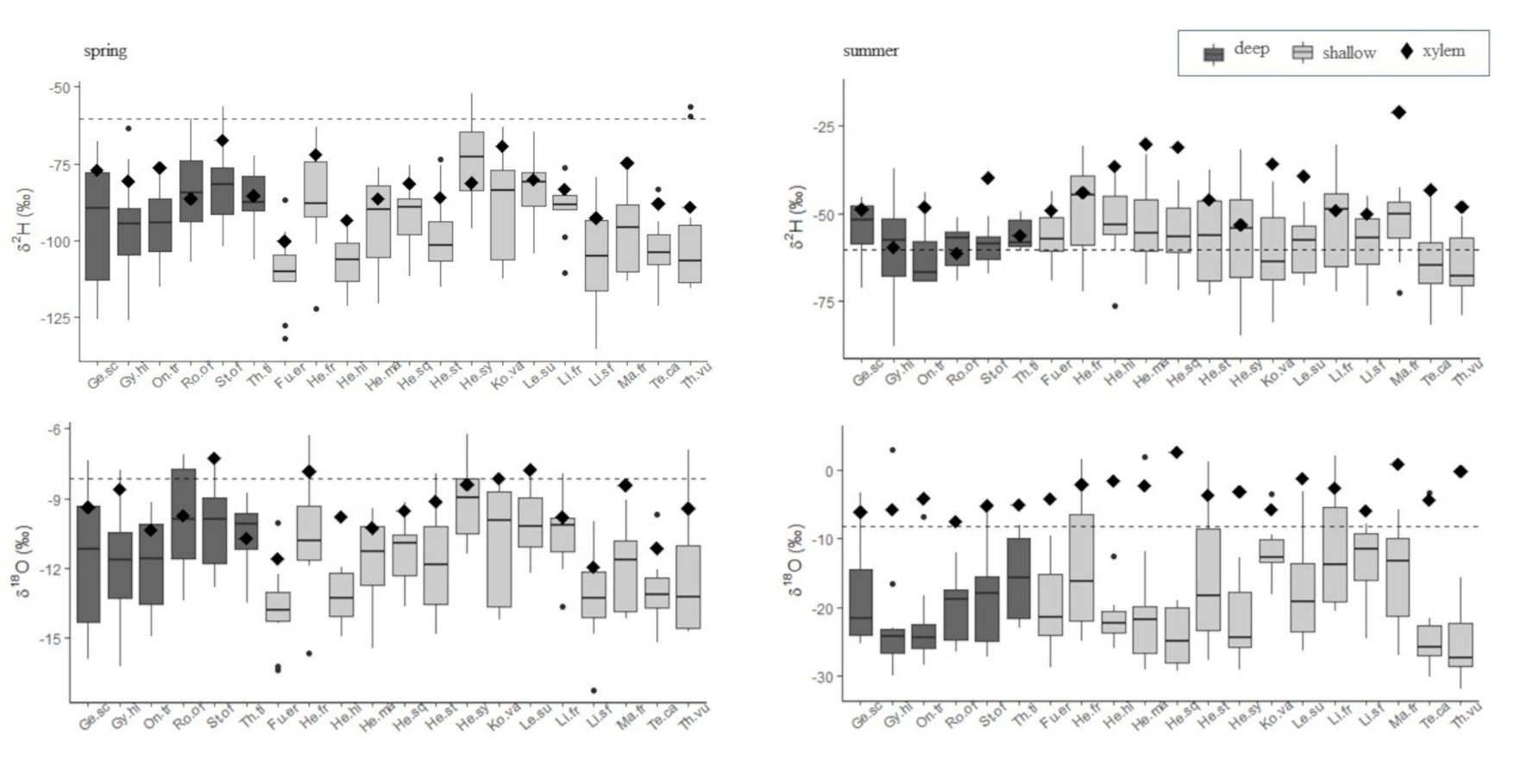
**Figure S6.** Spring and summer isotopic values of δ^2^H δ^18^O of free water in the soil underneath plants (boxes) and mean values for the xylem water of each species (diamonds). Boxes indicate the mean per species for the two soil depths analysed: 0-10 and 10-20 cm (*N* = 10 replicates), plus the upper and lower quartiles, and whiskers show maximum and minimum values. Darker boxes denote deep-rooted species; light grey boxes are shallow-rooted plants. Dashed line indicates groundwater values.

| Date | Precipitation (mm) | δ^2^H (‰) | δ^18^O (‰) |
| --- | --- | --- | --- |
| 10/2017 | 4 (36) | -21.40 (-42.56) | -2.29 (-6.21) |
| 11/2017 | 1 (30) | -5.84 (-61.15) | 2.49 (-8.89) |
| 12/2017 | 8 (21) | -46.96 (-51.55) | -6.19 (-7.49) |
| 01/2018 | 48 (21) | -93.22 (-60.15) | -12.68 (-8.32) |
| 02/2018 | 31 (22) | -69.82 (-61.41) | -10.47 (-8.46) |
| 03/2018 | 38 (19) | -57.36 (-43.16) | -7.44 (-6.40) |
| 04/2018 | 109 (39) | -111.33 (-39.93) | -14.81 (-5.85) |
| 05/2018 | 82 (44) | -34.79 (-31.47) | -4.97 (-4.70) |
| 06/2018 | 11 (26) | -25.98 (-31.27) | -3.09 (-4.42) |
| 07/2018 | 47 (17) | -29.34 (-24.60) | -4.39 (-3.60) |
| 08/2018 | 66 (17) | -25.63 (-22.68) | -4.37 (-3.56) |
| 09/2018 | 18 (30) | -20.53 (-30.62) | -3.61 (-4.71) |

**Table S1.** Water isotopic values of composite monthly samples of precipitation in Zaragoza in the hydrological year 2017-2018. As a reference, long-term means (1981-2010 for precipitation; 2000-2016 for isotopes) are shown between brackets. Isotope values were contributed by the REVIP (Red de Vigilancia de Isótopos en Precipitación), managed by CEDEX (Centro de Estudios de Técnicas Aplicadas del Centro de Estudios y Experimentación de Obras Públicas), in collaboration with AEMET (Agencia Estatal de Meteorología). Meteorological data were provided by AEMET OpenData (<https://opendata.aemet.es/>). It should be noted that precipitation recorded in August occurred after sampling.

**Table S2.** Statistics of GLMM analyzing the effects of the root depth, affinity for gypsum soils (gypsophily), season and their interaction on the isotopic composition (δ^18^O, δ^2^H, D-excess) of the xylem water of plants. Species, family and species nested within family were included as random terms. F-ratios and *p-*values are shown. Bold type indicates significant effects at *α* < 0.05.

| Factor | δ2H (‰) | | δ18O (‰) | | D excess (‰) | |
| --- | --- | --- | --- | --- | --- | --- |
|  | *F* | *p* | *F* | *p* | *F* | *p* |
| Gypsophily | 0.65 | 0.434 | 2.81 | 0.113 | 3.06 | 0.100 |
| Rootdepth | 0.10 | 0.333 | 3.86 | 0.070 | 6.88 | **0.021** |
| Season | 606.82 | **<0.001** | 305.50 | **<0.001** | 48.27 | **<0.001** |
| Gypsophily:rootdepth | 0.55 | 0.476 | 1.25 | 0.285 | 0.77 | 0.398 |
| Gypsophilly:season | 1.62 | 0.205 | 0.03 | 0.873 | 1.33 | 0.251 |
| Rootdepth:season | 23.95 | **<0.001** | 17.69 | **<0.001** | 6.03 | **0.015** |
| Gypsophily:rootdepth:season | 0.09 | 0.759 | 0.08 | 0.774 | 0.50 | 0.480 |

**Table S3.** Results of GLMMs analyzing the effects of root depth, affinity for gypsum soils (gypsophily) and their interaction on the isotopic composition (δ^18^O, δ^2^H, D-excess) of the xylem water of plants in spring and summer. Species, family and species nested within family were included as random terms. *F*-ratios and *p-*values are shown. Bold type indicates significant effects at *α* < 0.05.

|  |  | δ^2^H(‰) | | δ^18^O(‰) | | D-excess (‰) | |
| --- | --- | --- | --- | --- | --- | --- | --- |
|  |  | *F* | *p* | *F* | *p* | *F* | *p* |
| Spring | Gypsophily | 1.22 | 0.287 | 1.62 | 0.222 | 0.82 | 0.377 |
|  | Rootdepth | 0.25 | 0.628 | 0.00 | 0.981 | 2.47 | 0.138 |
|  | Gypsophily x rootdepth | 1.00 | 0.341 | 0.97 | 0.349 | 0.20 | 0.666 |
| Summer | Gypsophily | 0.09 | 0.768 | 1.48 | 0.244 | 1.81 | 0.200 |
|  | Rootdepth | 4.61 | **0.049** | 9.22 | **0.010** | 5.73 | **0.032** |
|  | Gypsophily x rootdepth | 0.25 | 0.626 | 0.71 | 0.419 | 0.49 | 0.499 |

**Table S4**. Results of GLMMs analyzing the effects of root depth in the isotopic composition of the soil water underneath the plants. Different analyses were run for each isotopeand season (two levels: spring and summer). Models included taxonomic family and species nested within family. *F* ratios and *p* values are shown. Bold type indicates significant effects at *α <0.05*

| **Season** | **Factor** | **Isotope** | ***F*** | ***p-value*** |
| --- | --- | --- | --- | --- |
| Spring | Gypsophily | δ^2^H | 0.51 | 0.487 |
|  |  | δ^18^O | 0.38 | 0.547 |
|  | Roothdepth | δ^2^H | 0.87 | 0.365 |
|  |  | δ^18^O | 0.88 | 0.364 |
|  | interaction | δ^2^H | 3.52 | 0.086 |
|  |  | δ^18^O | 3.59 | 0.084 |
| Summer | Gypsophily | δ^2^H | 0.13 | 0.727 |
|  |  | δ^18^O | 2.08 | 0.149 |
|  | Rootdepth | δ^2^H | 0.00 | 0.949 |
|  |  | δ^18^O | 0.47 | 0.501 |
|  | interaction | δ^2^H | 2.79 | 0.121 |
|  |  | δ^18^O | 1.30 | 0.275 |
